# First Evaluation after Implementation of a Quality Control System for the Second Line Drug Susceptibility Testing of *Mycobacterium tuberculosis* Joint Efforts in Low and High Incidence Countries

**DOI:** 10.1371/journal.pone.0076765

**Published:** 2013-10-11

**Authors:** Doris Hillemann, Sven Hoffner, Daniela Cirillo, Francis Drobniewski, Elvira Richter, Sabine Rüsch-Gerdes, the Baltic-Nordic TB- Laboratory Network, TB PAN-NET, ECDC ERLN-TB Networks

**Affiliations:** 1 National Reference Laboratory for Mycobacteria, Forschungszentrum Borstel, Borstel, Germany; 2 Supranational Reference TB Laboratory, Swedish Institute for Communicable Disease Control, Solna, Sweden; 3 Emerging Bacterial Pathogens, San Raffaele Scientific Institute, Milano, Italy; 4 National Mycobacterial Reference Laboratory, Public Health England, London, United Kingdom; McGill University, Canada

## Abstract

Three networks/projects involving 27 European countries were established to investigate the quality of second-line drug (SLD) susceptibility testing with conventional and molecular methods. 1. The “Baltic-Nordic TB-Laboratory Network” comprised 11 reference laboratories in the Baltic-Nordic States. They performed SLD testing in the first phase with a panel of 20 *Mycobacterium tuberculosis* strains. After several laboratories made technical changes a second panel of 10 strains with a higher proportion of resistant strains were tested. Although the concordance for Ofloxacin, Kanamycin, and Capreomycin was consistently high, the largest improvements in performance were achieved for the analysis of Ofloxacin resistant (from 88.9 to 95.0%), and Capreomycin resistant (from 71.0 to 88.9%) strains. 2. Within the FP7 TB PAN-NET project (EU Grant agreement 223681) a quality control panel to standardize the EQA (External Quality Assurance) for first-line drugs (FLD) and SLD testing for phenotypic and molecular methods was established. The strains were characterized by their robustness, unambiguous results when tested, and low proportion of secondary drug resistances. 3. The (European Reference Laboratory Network-TB) ERLN-TB network analyzed four different panels for drug resistance testing using phenotypic and molecular methods; in two rounds in 2010 the 31 participating laboratories began with 5 strains, followed by 10 strains and 6 additional crude DNA extracts in 2011 and 2012 were examined by conventional DST and molecular methods. Overall, we demonstrated the importance of developing inter-laboratory networks to establish quality assurance and improvement of SLD testing of *M. tuberculosis*.

## Introduction

The accurate determination of drug susceptibility is crucial for optimizing the treatment of tuberculosis (TB) and preventing transmission of drug resistant *M. tuberculosis* strains. However, there are methodological problems with current drug susceptibility testing (DST) of *M. tuberculosis*. Worldwide, no standardized DST methods for all SLDs exist. Laboratories perform tests using various methods, all based on the observation of inhibition of growth in media containing anti-tuberculosis drugs. In many countries the proportion methods on egg- or agar-based media are widely established, although the long turnaround time for obtaining results is a known disadvantage of these techniques. The DST methodologies for first-line drugs on solid media are mostly standardized and accepted [1], [2]. In order to improve turn-around times numerous new techniques have been introduced. Growth detection based on the measurement of CO_2_ production (BACTEC 460TB, Becton Dickinson Microbiology Systems, Sparks, MD [BD] and the MB/BacT system, bioMérieux, Marcy l'Etoile, France) or oxygen consumption (mycobacterial growth indicator tube system, BACTEC MGIT 960, [BD]) have been developed. Although a variety of methods exist, the majority have been replaced by the MGIT 960 method, since it is more versatile, nonradiometric, and automated. The method has been widely validated for the reliable and rapid testing of first-line (and some second-line) drug susceptibilities of *M.tuberculosis* isolates [3]–[6]. The emergence of drug-resistant strains led to the increased introduction of SLDs for tuberculosis treatment and subsequently to the need to extend current methods to SLD susceptibility testing [7]. Whereas the critical concentrations for SLD with BACTEC 460 were assessed more than ten years ago [8], comparable studies were accomplished for MGIT more recently [9], [10]. In 2008, during an WHO Expert Committee meeting in Geneva, critical concentrations were specified in interim guidelines, which are recommended to use for SLD [2].

The WHO/IUATLD Supranational Reference Laboratory (SRL) network was created in 1994, to validate the accuracy of the DST methods used in laboratories across the world and support global drug resistance surveillance. Up until 2011, 28 WHO Supranational Reference Laboratories (SRLs) had actively participated in 18 rounds of proficiency testing including FLD, and since 2007 (14^th^ round) also SLD demonstrating the success of the network in producing a substantial improvement in performance [11].

Although a functioning supranational network for DST exists, proficiency testing especially for SLD was for a long time neglected in both high and low income countries. In recent years, three networks/projects were initiated to establish SLD testing with conventional and molecular methods: the “Baltic-Nordic TB-Laboratory Network” [12], the network in the WP3 of the TB PAN-NET project (EU Grant agreement 223681), and the ECDC *(European Centre for Disease Prevention and Control)* ERLN-TB network, which was founded to generate a European proficiency testing system for all mycobacterial diagnostic procedures. By summarizing results and experiences from all three networks we aimed to give an overview of current External Quality Assurance (EQA) of SLD and the reliability of SLD results in reference laboratories in the European region.

## Materials and Methods

### Study Design “Baltic-Nordic TB-Laboratory Network”

Overall 11 laboratories participated in the study; all were members of the “Baltic-Nordic TB-Laboratory Network”, which was created in 2001, primarily to ensure safe working conditions for DST. The study was carried out in two phases: **Phase I.** This phase was designed to compare the SLD susceptibility testing results with a panel of 20 strains chosen by the NRC in Borstel, Germany. All laboratories were free in their choice of which drugs they wanted to test and DST methods used. In order to minimize the risk of handling extensively resistant strains, the majority of strains were drug susceptible with only a few exhibiting resistance. Results were reported to Borstel, analyzed and discussed with all participants. **Phase II.** In the second phase a set of 10 strains was chosen and sent to the participants. The second panel comprised proportionally more resistant strains than the first panel. In this phase laboratories were allowed to test the strain repeatedly before reporting the results. SLD testing results were again analyzed and compared to results in phase I. A detailed questionnaire was sent out requesting details of the respective methods that were performed in the laboratories.

### Participating Laboratories, Test Methods and Method of Analysis

In each round 10 laboratories participated (9 were the same in both rounds). The laboratories tested a maximum of 7 drugs, (Amikacin, Capreomycin, Cycloserine, Ethionamide/Protionamide, Kanamycin, Ofloxacin, PAS). The following methods were applied: the proportion method on Löwenstein-Jensen medium, and the critical concentration method on 7H10 medium, BACTEC 460 and MGIT 960 [13]–[15] media.

For the assessment of SLD susceptibility testing, results from all laboratories and drugs used were included in the analysis. None of the laboratories were chosen as an arbiter that defines the “true” result of resistance. A strain was defined as “susceptible or “resistant”, based on the majority decision of laboratories, when reporting the result of the strain. In the case of an equal score, the respective strains were reanalyzed in Borstel by two alternative methods and this result was applied. In a second step, the concordance between results was estimated by the percentage of laboratories having obtained this result.

### Study Design “Work Package 3 of the TB PAN-NET”

Overall 14 partner laboratories participated. Laboratories were free to choose which methods to apply on any of the sent EQA panels. In the first round a panel of 20 susceptible and resistant strains were sent for FLD and SLD testing together with a questionnaire to complete with the respective methods that were performed in the laboratories. In an intermediate analysis some laboratories investigated MIC levels of strains with doubtful results. In the second round of panel testing 20 laboratory generated strains were used for FLD and SLD conventional DST. Some groups additionally performed molecular-based methods. The results of both rounds were used to create a selection of robustly characterized strains.

### Study Design “ECDC ERLN-TB Network”

Overall 31 laboratories participated in the study; all ensured safe working conditions and were free to participate in any of the sent EQA panels. The study was carried out in four phases. Until now, four different rounds of quality control were accomplished. Round 1 in spring 2010 included 5 strains for FLD DST. In autumn 2010, SLD testing with phenotypic and molecular methods were added. In autumn 2011 and 2012 a further 10 strains and 6 crude DNA extracts for FLD and SLD testing with phenotypic and molecular methods were added.

All participants received an individual analysis of their performance and a certificate summarizing the score obtained.

## Results

### “Baltic-Nordic TB-Laboratory Network”: Phase I

20 strains were analyzed by 10 laboratories. All reported results were included except “borderline” and “no growth”. The level of concordance between the results was calculated (Table 1, upper part, Phase I) and was higher between strains that were assessed “susceptible” compared to those that were assessed “resistant”. The highest levels of concordance were found for Kanamycin and Ofloxacin with 98.3% and 98.9%, respectively. The lowest level of concordance was found for Ethionamide at 82.9% and PAS at 92.0%, respectively. In order to improve the quality of SLD various technical changes were implemented by members of the network: (Table 2) **Amikacin**: One laboratory changed the critical concentration from 4 to 1 µg/ml, two others from 2 to 1 µg/ml and used BACTEC MGIT 960 instead of BACTEC 460. **Capreomycin**: Some laboratories switched from Capreomycin to Capreomycin sulphate and changed the drug concentration from 5 to 1.25 µg/ml in BACTEC 460 and from 1.25 to 2.5 µg/ml now using the BACTEC MGIT 960 instead of the BACTEC 460 system. Instead of tap water deionised water was used as diluent of the drug in one laboratory. **Cycloserine**: Although the substance used was obtained from different companies, all used D-cycloserine. All laboratories, except one which used Middlebrook 7H10 media had a slightly higher concentration of Cycloserine (40 µg/ml) as critical concentration and revealed a lower number of resistant strains than those, who used the lower 30 µg/ml concentration. Nevertheless, the concentrations of D-Cycloserine were retained. **Ethionamide/Protionamide**: One laboratory switched from methanol to ethylene glycol, another from ethylene glycol to DMSO as the solvent, and two switched from the BACTEC 460 to BACTEC MGIT 960 method. Another laboratory reduced the drug concentrations tested from 2, 4, and 6 to only the recommended critical concentration (Ethionamide 5 µg/ml; Protionamide 2.5 µg/ml). **Kanamycin**: Kanamycin is delivered as mono-sulphate or di-sulphate, but most used the mono-sulphate. The calculation of initial weight of Kanamycin has to be done carefully in the case of different substances. Since there was total concordance with a drug concentration of 5 µg/ml and water as solvent and diluent no changes were made. **Ofloxacin**: Four different solvents were used for Ofloxacin: water, 0.1 m NaOH, DMSO, and propylene glycol. One laboratory changed the drug concentration from 1 to 2 µg/ml using BACTEC MGIT 960. Another reduced the drug concentrations tested from 2, 4, and 6 to only 2 µg/ml. **PAS**: The critical drug concentration was reduced in two cases from 4 to 1 and 2, and from 4 to 0.5 together with a change from using the BACTEC 460 to the Löwenstein Jensen media. Another laboratory reduced the drug concentrations tested from 2, 4, and 6 using the BACTEC MGIT 960 to a single concentration of 4 µg/ml.

**Table 1:** Agreement of SLD testing in different laboratories in phase I and phase II of the “Baltic-Nordic TB-Laboratory Network” study.

		Concordance % (no. of strains)						
	Strains	Amikacin	Capreomycin	Cycloserine	Ethionamide/Protionamide	Kanamycin	Ofloxacin	PAS
Phase I								
	susceptible	95.3 (17)	97.7 (16)	96.4 (20)	87.9 (14)	100 (16)	99.4 (19)	92.6 (19)
	Resistent	84.7 (3)	71.0 (4)	No strains	71.3 (6)	91.7 (4)	88.9 (1)	80.0 (1)
	**Total**	**93.8 (20)**	**92.3 (20)**	**96.4 (20)**	**82.9 (20)**	**98.3 (20)**	**98.9 (20)**	**92 (20)**
Phase II								
	susceptible	86.7 (5)	100 (6)	91.8 (7)	89.1 (6)	93.3 (5)	100 (6)	97.5 (5)
	resistent	60.0 (5)	88.9 (4)	66.7 (3)	81.9 (4)	96.7 (5)	95 (4)	91.8 (5)
	**Total**	**71.1 (10)**	**95.6 (10)**	**84.3 (10)**	**86.2 (10)**	**95.0 (10)**	**98.0 (10)**	**94.6 (10)**

PAS, p-amino salicylic acid.

**Table 2:** Methods applied in the different laboratories in both phases of SLD proficiency testing (“Baltic-Nordic TB-Laboratory Network” study).

	Drug concentration in µg/ml (number of laboratories*)				
Drug	Proportion method	Critical concentration	BACTEC	MGIT	MIC
Amikacin	20+40 (2)	6 (1), 8 (1)	1(6)	1(3)	
Capreomycin	20+40 (2)	2 (1)	1.25 (5), 5 (1)	2.5 (3)	
Cycloserine	30 (5); 20+40 (1)	40 (1)			30, 40 (1)
Ethionamide/Protionamide	20+40 (1);		2.5 (3), 5 (2)	2.5 (3)	4 (1)
Kanamycin	20+40 (2)		5 (3)	5 (3)	
Ofloxacin	0.5+1+2 (1)	2.5 (1)	2(6)	1 (1), 2 (2)	4 (1)
PAS	0.25 (1); 0.5 (2); 0.5+1(1)		1+2(1); 2(1); 4(1)	4 (1)	2 (1)

* some laboratories used more than one method.

PAS, p-amino salicylic acid.

LJ, Löwenstein Jensen; MIC, minimal inhibitory concentration.

### “Baltic-Nordic TB-Laboratory Network”: Phase II

Based on the good results of phase I, in the second panel the proportion of resistant strains was increased considerably from a mean of 13.6% to 41.4%. Best results were again achieved with Ofloxacin testing (Table 1). Likewise an enhancement was obtained for Capreomycin, Ethionamide/Protionamide and PAS. For Amikacin and Cycloserine the results were poorer for both susceptible and resistant strains.

### The “WP3 of the TB PAN-NET” Project

**Figure 1:**
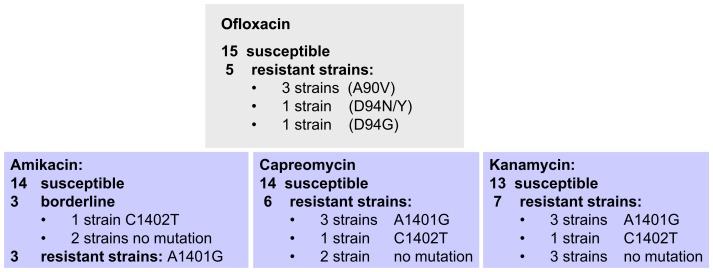
Compilation of strains in round 1 of the WP3 of the TB PAN-NET” project.

In round 1, 13 laboratories agreed to participate in the proficiency testing of a panel of 20 strains, which were laboratory generated monoresistant strains (or polyresistant but sensitive to first line drugs) to second line drugs (5 Fluoroquinolone resistant, 7 Kanamycin resistant, 6 Capreomycin resistant, from which 3 were also Amikacin resistant and 3 borderline) (Figure 1). Susceptibility of all 20 strains to FLD was confirmed by some laboratories. Phenotypic and molecular testing of Fluoroquinolone resistance showed 100% concordance between all laboratories (5 resistant and 15 susceptible strains). Detection of resistance related mutations of the injectable drugs Amikacin, Capreomycin, and Kanamycin was also 100% concordant, whereas the phenotypic testing showed unambiguous results only for Capreomycin (6 resistant and 14 susceptible) and Kanamycin (7 resistant and 13 susceptible). Regarding Amikacin, 3 strains (with no mutation in the rrs gene) were identified as susceptible, mainly when using the proportion method on solid medium, but resistant when mainly using the MGIT liquid system. Two laboratories confirmed with both methods the respective “susceptible” and “resistant” results. One strain with the rrs mutation C1402T showed also disconcordant results for Amikacin. In order to clarify how these strains should be classified, the doubtful strains were retested and underwent a MIC analysis. All strains showed elevated MIC levels compared to the H37Rv reference strain, with the critical concentration slightly lower (<40 µg/ml) when using the proportion method on LJ medium and at the critical concentration (1 µg/ml) with the MGIT method. We observed that the strains showed elevated MIC levels dependant on the age of the subculture. Since the laboratories agreed upon the fact that for these 4 strains an unambiguous result could not be given, they were classified as borderline but excluded from the quality control panel. In order to generate a greater number of strains monoresistant to SLD or polyresistant (but not MDR), a second round of 20 strains were prepared “in vitro” and tested. Again, the Fluoroquinolone testing revealed a high interlaboratory concordance rate (99.5%, only 1 false resistant result). Kanamycin testing of the panel also showed a high concordance rate (98.3%, only 2 false susceptible results). Capreomycin testing however was again less concordant with 2 strains and Amikacin testing with 3 strains showing ambiguous results; these were excluded from the quality control panel. From both rounds of proficiency testing the selection of robustly characterized strains for SLD DST was finalized (Table 3).

**Table 3:** Agreement of SLD results between laboratories in the “Workpackage 3 of the FP7 TB PAN-NET”.

Concordance % (no. of strains tested, no of tests performed*)				
	Molecular methods**		Phenotypic methods***	
Drug tested	Susceptible strains	Resistent strains	Susceptible strains	Resistent strains
Ofloxacin	100% (40, 490)	100% (15, 185)	99.8% (40, 490)	100% (185)
Amikacin	100% (47, 577)	100% (9, 111)	99.8% (39, 469)	100% (9, 111)
Capreomycin	100% (44, 540)	100% (12, 148)	98.5% (37, 327)	95.2% (17, 209)
Kanamycin	100% (44, 540)	100% (12, 148)	98.7% (38, 458)	96.7% (18, 139)

* All strains were tested in three rounds of testing: round 1 (n = 13), in the intermediate round (n = 11), and in round 2 of the “Workpackage 3 of the FP7 TB PAN-NET”.

** All laboratories applied line probe assays, some additionally DNA sequencing methods.

*** The majority of laboratories applied MGIT 960 DST, some the proportion method on solid media, but the data are incomplete; intermediate level strains were excluded from this analysis.

### “ECDC ERLN-TB Network”

**Figure 2:**
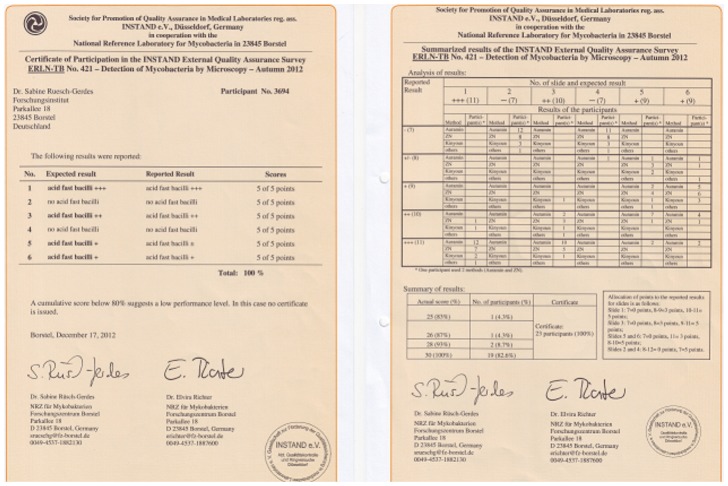
Certificates issued for a succesful EQA round.

All participating laboratories received an individual analysis with a certificate (see example Figure 2). In the first round in spring 2010 only FLD were tested showing high concordance rates between the 31 participants (Table 4): Isoniazid (100%), Rifampicin (98.1%), Ethambutol (99.4%), Pyrazinamide (98.2%), and Streptomycin (99.3%). In the second to fourth round in autumn 2010, 2011 and 2012, FLD and SLD were tested again with high concordance rates between 23, 25, and 23 participants, respectively (Table 4): Isoniazid (98.2%, 99.6%, and 100%), Rifampicin (97.4%, 100%, and 99.1%), Ethambutol (96.5%, 97.4%, and 99.5%), Pyrazinamide (86.7%, 94.0%, and 97.6%), Streptomycin (95.5%, 98.3%, and 98.8%), Fluoroquinolones (100%, 100%, and 98.1%), Amikacin (100%, 96.1%, and 99.4%), and Capreomycin (94.5%, 100%, and 98.2%).

**Table 4:** Agreement of SLD results between laboratories in the four rounds of quality control.

	Concordance % (no of tests performed*)									
	Spring 2010		Autumn 2010		Autumn 2011		Autumn 2012		Total	
Drug	susc. strains**	res.strains**	susc. strains	res.strains	susc. strains	res.strains	susc. strains	res.strains	susc. strains	res.strains
Isoniazid	100 (124)	100 (31)	97.8 (90)	100 (23)	99.4 (174)	100 (75)	100 (184)	100 (46)	99.5 (572)	100 (175)
Rifampicin	98.9 (93)	96.8 (93)	97.8 (92)	95.5 (93)	100 (174)	100 (75)	99.5 (207)	95.5 (22)	99.3 (566)	97.8 (181)
Ethambutol	99.4 (154)	n.d.	96.5 (114)	n.d.	98.6 (216)	84.2 (19)	99.5 (207)	n.d.	98.7 (691)	84.2 (19)
Pyrazinamide	97.7 (88)	100 (22)	86.7 (75)	n.d.	94.4 (197)	90 (20)	98.9 (188)	85.0 (20)	95.4 (548)	91.9 (62)
Streptomycin	99.2 (119)	100 (30)	97.1 (69)	90 (20)	99.5 (190)	93.8 (48)	98.6 (220)	99.2 (119)	98.8 (598)	97.2 (217)
Ofloxacin	n.d.***	n.d.	100 (65)	n.d.	100 (161)	100 (17)	98.6 (144)	94.1 (17)	99.5 (370)	97.1 (34)
Amikacin	n.d.	n.d.	100 (53)	n.d.	96.1 (179)	n.d.	100 (136)	97.1 (34)	98.1 (368)	97.1 (34)
Capreomycin	n.d.	n.d.	96.4 (55)	n.d.	100 (158)	n.d.	100 (136)	91.2 (34)	99.4 (349)	91.2 (34)
Kanamycin	n.d.	n.d.	n.d.	n.d.	100 (116)	76.9 (13)	100 (96)	95.5 (22)	100 (212)	88.6 (35)

* The majority of laboratories applied MGIT 960 DST, only one laboratory performed the proportion method on solid media, but the data are incomplete.

** susc. = susceptible, res. = resistant; intermediate level strains were excluded from this analysis.

*** n.d. = not done.

Certificates were issued for all 31 participants in spring 2010, 21 of 23 (91.3%) in autumn 2010, 24 of 25 (96%) in autumn 2011, and all 23 (100%) in autumn 2012. Despite the high summarized concordance rates, deviations were found with false susceptible results highest with Ethambutol (15.8%), Kanamycin (11.4%), and Pyrazinamide (8.1%), and false resistant results highest with Pyrazinamide (5.6%) (Table 4).

Altogether three different panels were analyzed with molecular methods by between18 and 26 participants, using 5 crude DNA extracts in autumn 2010, and 10 strains plus 6 additional DNAs in autumn 2011 and 2012. The majority of laboratories used line probe assays (95% in 2010, 100% in 2011, and 96% in 2012, with only a few applying DNA sequencing alone or in combination, and additionally one laboratory a PCR amplification-restriction analysis). In comparison to phenotypic methods, the concordance rates were even higher with 99.0% for Isoniazid (n = 88 participants in 4 rounds), 99.1% for Rifampicin (n = 87), 99.3% for Ethambutol (n = 66), 100% for Pyrazinamide (n = 2), 88.9% for Streptomycin (n = 2), 99.7% for Flouroquinolones (n = 64), and 99.6% for Amikacin and Capreomycin (n = 63). Rates of false resistant results were correspondingly low ranging from 0.3% (Amikacin, Capreomycin, Ethambutol) to 1.5% (Isoniazid), and false susceptible results ranged from 1% (Isoniazid) to 1.5% (Rifampicin). Calculation of false resistant and susceptible results was excluded for antibiotics for which only a low number of resistant strains were tested or few participants sent results.

## Discussion

Laboratory services have an important role in improving the delivery of health care and reducing the prevalence of TB, particularly drug resistant TB. Quality control and proficiency testing are fundamental tools that ensure accuracy in laboratories by comparing quality to other laboratories, evaluating the general performance level of the laboratory’s service, and detecting laboratory facilities with unacceptable levels of proficiency. This concept culminated in the establishment of the current supranational network of the WHO/IUATLD with their external quality assurance system.

In the present multicenter study one aim was to establish a network as the basis for high quality accurate SLD susceptibility testing. Our second goal was to assess whether we, as a network, could improve the reliability of DST results. Based on the experience with FLD it is known that continued proficiency testing with exchange of information and technical assistance has led to improved DST quality of participating laboratories [16].We observed a similar improvement in performance in SLD testing by participant laboratories.

The highest improvements were found in the **Baltic-Nordic TB-Laboratory Network** study from round I to round II resulting in a rise in the concordance rates in the category of resistant strains for Ofloxacin (from 88.9 to 95.0%), Kanamycin (from 91.7 to 96.7%), and Capreomycin (from 71.0 to 88.9%) testing. Within the other networks the improvement was smaller but started from a higher base-line of accuracy.

One of the major outcomes from these studies was the finding that the reliability of phenotypical SLD depends on the drug tested. As already well known for FLD differences in DST depend on the drug compound used. Isoniazid and Rifampicin resistance can be reliably measured; resistance to Pyrazinamide, Ethambutol, and Streptomycin is more difficult [11], [17]–[19]. Long-term studies undertaken in 1994–2002 revealed average sensitivities to detect resistance to Isoniazid of 98.7% and to Rifampicin 97.2% [20]. Although in our studies the number of samples is not comparable to the many rounds of proficiency testing of FLD over a long time period, we could show that for Ofloxacin, Kanamycin and Capreomycin a concordance of >95% could be achieved. In Italy also a pilot round of SLD in 2010 showed similar results with >95% specificity and efficiency [21]. The analysis of 57733 tests of DST for FLD in the U.S from 1994 to 2008 yielded a significantly higher agreement of susceptible strains (98.4%) compared to resistant strains (91%) [22]. This can also be seen by comparison of phase I and II in the **Baltic-Nordic TB-Laboratory Network** study (Table 1) with decreased concordance rates in the second phase, where the proportion of resistant strains was higher. There was general agreement among participants for biosafety reasons to avoid sending highly drug resistant strains for quality control purposes around the world. Consequently, we developed a selected panel of *in vitro* generated resistant strains with single or poly drug resistance to FLD and SLD but without MDRTB. This panel was tested, characterized and a selection of reliable strains was made. There was a discussion as to whether the more problematic strains should be used, since a laboratory has to deal with borderline results as well. In our opinion only unambiguous strains (susceptible or resistant) should be used, since the overall improvement of quality was the main goal, which can be done most easily with clear defined strains.

We are convinced that second line DST results can be considered as reliable and comparable to some FLD. Furthermore we now have the capacity to reliably identify XDR strains (multidrug resistant strains with additional resistance to a Fluoroquinolone and an injectable drug).

Noteworthy was the high concordance of results with molecular methods, although these samples were tested for the first time. The reason for this may be that the molecular methods are less dependent on personal handling than conventional DST. Sources of errors, such as sample mix up or technical errors may occur with both molecular and microbiological methods, but factors like the critical inoculum size for example require experienced technicians to reduce variability for microbiological DST methods.

The long-term objective of the projects was to set up a functioning network to improve and control the quality of DST for SLDs in all participating countries. The main factor for influencing the success of this network was the exchange of information between the project partners to rule out major technical problems such as usage of the proper solvent and concentration of the drugs to be tested.

The networks included laboratories from more than 30 countries. It can serve as a model system for other unconnected countries and all laboratories which want to participate in a system that has proven to be efficient in proficiency testing.

Until now INSTAND offers only testing for microscopy, primary culture, culture differentiation, Nucleic Acid Amplification Tests (NAAT), and FLD, but this may be expanded with SLD and the detection of resistance related mutations by molecular methods. If the decision is made to include new methods for quality control, the newly developed laboratory-generated strains may serve as a basis of strains having the advantages of being well characterized, no mixed populations, and safe due to single drug resistances.

With the multicenter studies presented here, we have shown that it is possible to produce reliable results for many SLDs between laboratories applying different analytical methods. The application of molecular methods also yielded a high degree of interlaboratory concordance. The joint correction and adjustment of techniques yielded an improvement of quality in laboratories in many countries.

## References

[1] Woods GL, Brown-Elliott BA, Desmond EP, Hall GS, Heifets L, et al (2003) Susceptibility testing of mycobacteria, nocardia, and other aerobic actinomycetes; approved standard. NCCLS 23: M24–A. 31339680

[2] Barrera L, Cooreman E, de Dieu Iragena J, Drobniewski F, Duda P, et al.. (2008) Policy guidance on drug-susceptibility testing (DST) of second-line antituberculosis drugs. WHO/HTM/TB/2008.392. 26290924

[3] Garrigó M, Aragón LM, Alcaide F, Borrell S, Cardeñosa E, et al (2007) Multicenter laboratory evaluation of the MB/BacT Mycobacterium detection system and the BACTEC MGIT 960 system in comparison with the BACTEC 460TB system for susceptibility testing of *Mycobacterium tuberculosis* . J Clin Microbiol 45: 1766–1770. 17442793 10.1128/JCM.02162-06 PMC1933104

[4] Pfyffer GE, Palicova F, Rüsch-Gerdes S (2002) Testing of susceptibility of *Mycobacterium tuberculosis* to pyrazinamide with the nonradiometric BACTEC MGIT 960 system. J Clin Microbiol 40: 1670–1674. 11980940 10.1128/JCM.40.5.1670-1674.2002 PMC130957

[5] Rüsch-Gerdes S, Domehl C, Nardi G, Gismondo MR, Welscher H-M, et al (1999) Multicenter evaluation of the mycobacterial growth indicator tube for testing susceptibility of *Mycobacterium tuberculosis* to first-line drugs. J Clin Microbiol 37: 45–48. 9854062 10.1128/jcm.37.1.45-48.1999 PMC84164

[6] Scarparo C, Ricordi P, Ruggiero G, Piccoli P (2004) Evaluation of the fully automated BACTEC MGIT 960 system for testing susceptibility of *Mycobacterium tuberculosis* to Pyrazinamide, Streptomycin, Isoniazid, Rifampin, and Ethambutol and comparison with the radiometric BACTEC 460 method. J Clin Microbiol 42: 1109–1114. 15004061 10.1128/JCM.42.3.1109-1114.2004 PMC356895

[7] World Health Organization (2004) Anti-tuberculosis drug resistance in the World. Report No. 3. The WHO/IUATLD global project on anti-tuberculosis drug resistance surveillance. WHO/HTM/TB/2004.343.

[8] Pfyffer GE, Bonato DA, Ebrahimzadeh A, Gross W, Hotaling J, et al (1999) Multicenter laboratory validation of susceptibility testing of *Mycobacterium tuberculosis* against classical second-line and newer antimicrobial drugs by using the radiometric BACTEC 460 technique and the proportion method with solid media. J Clin Microbiol 37: 3179–3186. 10488174 10.1128/jcm.37.10.3179-3186.1999 PMC85522

[9] Kruuner A, Yates MD, Drobniewski FA (2006) Evaluation of MGIT 960-based antimicrobial testing and determination of critical concentrations of first- and second-line antimicrobial drugs with drug-resistant clinical strains of *Mycobacterium tuberculosis* . J Clin Microbiol 44: 811–818. 16517859 10.1128/JCM.44.3.811-818.2006 PMC1393078

[10] Rüsch-Gerdes S, Pfyffer GE, Casal M, Chadwick M, Siddiqi S (2006) Multicenter laboratory validation of the BACTEC MGIT 960 technique for testing susceptibilities of *Mycobacterium tuberculosis* to classical second-line drugs and newer antimicrobials. J Clin Microbiol 44: 688–692. 16517840 10.1128/JCM.44.3.688-692.2006 PMC1393114

[11] Laszlo A, Rahman M, Raviglione M, Bustreo F (1997) Quality assurance programme for drug susceptibility testing of Mycobacterium tuberculosis in the WHO/IUATLD Supranational Laboratory Network: first round of proficiency testing. Int J Tuberc Lung Dis 1: 231–238. 9432369

[12] Johansen IS, Larsen AR, Sandven P, Petrini B, Soini H, et al (2003) Drug susceptibility testing of *Mycobacterium tuberculosis* to fluoroquinolones: first experience with a quality control panel in the Nordic-Baltic collaboration. Int J Tuberc Lung Dis 7: 899–902. 12971676

[13] Kent PT, Kubica G (1985) Public Health Mycobacteriology: A guide for the level III laboratory. Atlanta, GA: US Department of Health and Human Services, Centers for Disease Control 1985: 159–184.

[14] Canetti G, Fox W, Khomenko, Mahler HT, Menon NK, et al (1969) Advances in techniques of testing mycobacterial drug sensitivity and the use of sensitivity tests in tuberculosis control programmes. Bull World Health Organ 41: 21–43. 5309084 PMC2427409

[15] Siddiqi SH (1995) BACTEC 460 TB System. Product and procedure manual, revision D. Becton Dickinson Diagnostic System, Sparks, MD.

[16] Fattorini L, Iona E, Cirillo D, Migliori GB, Orefici G, et al (2008) External quality control of *Mycobacterium tuberculosis* drug susceptibility testing: results of two rounds in endemic countries. Int J Tuberc Lung Dis 12: 214–217. 18230256

[17] Shulgina MV, Malakhov VN, Hoffner SE, Haile M, Wright A (2009) Results of the external quality assessment of *Mycobacterium tuberculosis* drug susceptibility testing in Russia, 2005–2007. Int J Tuberc Lung Dis 13: 1294–1300. 19793436

[18] Laslo A, Rahman M, Espinal M, Raviglione M (2002) World Health Organization/International Union Against Tuberculosis and Lung Disease Network of Supranational Reference Laboratories. Quality assurance programme for drug susceptibility testing of *Mycobacterium tuberculosis* in the WHO/IUATLD Supranational Reference Laboratory Network: five rounds of proficiency testing,1994–1998. Int J Tuberc Lung Dis 6: 748–756. 12234129

[19] Bai G-H, Kim S-J, Chang CL (2007) Proficiency analysis of drug susceptibility testing by national-level tuberculosis reference laboratories from 1995 to 2003. J Clin Microbiol 45: 3626–3630. 17855570 10.1128/JCM.00784-07 PMC2168479

[20] Kim SJ (2005) Drug-susceptibility testing in tuberculosis: methods and reliability of results. Eur Respir J 25: 564–569. 15738303 10.1183/09031936.05.00111304

[21] Fattorini L, Migliori GB, Cassone A, Mustazzolu A, Piccaro G, et al (2012) Italian Multicentre Study on Resistance to Antituberculosis Drugs Group. Proficiency testing of first- and second-line anti-tuberculosis drugs in Italy. Eur Respir J 39: 1263–1266. 22547735 10.1183/09031936.00129011

[22] Angra PK, Taylor TH, Iademarco MF, Metchock B, Astles JR, et al (2012) Performance of tuberculosis drug susceptibility testing in U.S. laboratories from 1994 to 2008. J Clin Microbiol 50: 1233–1239. 22301024 10.1128/JCM.06479-11 PMC3318540

